# Impact of Duodopa on Quality of Life in Advanced Parkinson's Disease: A UK Case Series

**DOI:** 10.1155/2013/362908

**Published:** 2013-02-13

**Authors:** T. Foltynie, C. Magee, C. James, G. J. M. Webster, A. J. Lees, P. Limousin

**Affiliations:** ^1^Sobell Department of Motor Neuroscience, UCL Institute of Neurology, Queen Square, London WC1N 3BG, UK; ^2^National Hospital for Neurology and Neurosurgery, Queen Square, London WC1N 3BG, UK; ^3^Department of Gastroenterology, University College Hospital NHS Trust, 235 Euston Road, London NW1 2BU, UK; ^4^Reta Lila Weston Institute, 1 Wakefield Street, London WC1N 1PJ, UK

## Abstract

Treatment options in advanced Parkinson's disease (PD) include subcutaneous apomorphine, pallidal or subthalamic nucleus Deep Brain Stimulation (DBS), or levodopa/carbidopa intestinal gel (LCIG/Duodopa). In this study, we describe the outcome of 12 PD patients with PD related complications started on LCIG, with respect to their quality of life measured by a disease specific validated scale—the PDQ39, together with diaries recording time spent “On,” “Off,” “Dyskinetic,” or “Asleep.” At the time of latest follow up, improvements were observed in both the PDQ39 Summary index as well as diary reports of PD symptom control following introduction of LCIG, supporting its use in well selected patients. The use of a trial period of LCIG via naso-jejunal administration allows objective evaluation of improvement in PD symptom control in advance of the placement of the more invasive percutaneous jejunostomy procedure. The decision to embark on LCIG, apomorphine or DBS should be supported by input from centres with experience of all 3 approaches. Since LCIG is an expensive option, development of the most appropriate future commissioning of this therapy in the absence of Class 1 evidence requires careful scrutiny of the outcomes of its use in a broad range of published series.

## 1. Introduction

One of the key outcomes for the UK National Health Service Outcomes Framework 2012/2013 is “Enhancing the quality of life for people with long term conditions.” Neurodegenerative diseases including Parkinson's disease (PD) represent a major target area for outcome improvement. While the majority of patients with Parkinson's disease respond well to conventional oral medication, some patients (particularly those with young onset PD) survive for many decades with the illness and, in the later or “advanced” stages, experience severe disabling complications emerging from the combination of advancing degeneration and chronic medication use. These complications consist of severe adventitious involuntary movements often referred to as L-dopa-induced dyskinesias, and unpredictable “On/Off” fluctuations, necessitating the use of other strategies to maintain acceptable quality of life.

 Oral and transdermal anti-Parkinsonian therapy fails to provide sufficient relief from the symptoms of advanced PD in a significant proportion of patients, in whom consideration should be given regarding the use of subcutaneous apomorphine, subthalamic or pallidal Deep Brain stimulation (DBS), or levodopa/carbidopa intestinal gel (LCIG/Duodopa). Patients reaching the advanced stages of PD while still relatively young and free from other comorbidities will frequently actively seek out these therapies and are often (although not exclusively) better candidates for their use. However the use of all 3 of these approaches is relatively expensive, and thus in the context of the National Health Service in the United Kingdom, their use requires authorisation via “Individual Funding Requests.” For the current use of LCIG, each respective patient must be judged to meet criteria for “Exceptional Circumstances” and in the absence of National guidelines, the evidence supporting the efficacy of LCIG is evaluated by local committees before funding is agreed.

The use of enteral L-dopa infusion in the treatment of advanced PD has developed over the last 20 years [1, 2]. LCIG has previously been shown in 2 small crossover design trials to increase the proportion of the day patients spent in the ON state, with reduction in Levodopa-induced dyskinesias (LID) and improvement in quality of life when compared to oral Levodopa [3, 4], confirmed in a double blind, double dummy trial of 71 patients with a 12-week followup [5]. Open label series have also confirmed that the beneficial effects of LCIG are maintained in the long term [6–9], can be maintained using continuous 24 hour/day LCIG exposure [4], and also have benefit on nonmotor symptoms [10]. Nevertheless the majority of reports with a long-term followup inevitably include small numbers of patients.

There is therefore an appropriate level of caution regarding the widespread authorisation of LCIG use in many countries in view of the expense of the drug (each cassette costs *£*77; therefore, the typical use of 1 cassette per day equates to an annual treatment cost of *£*28 105) that must be weighed against competing needs for healthcare provision in this same and other patient groups. Furthermore the percutaneous gastrostomy and jejunal extension tubes required for LCIG therapy have been associated with frequent complications in PD patients [9], and there are emerging concerns regarding an apparent increased frequency of neuropathy among patients receiving LCIG [11].

In view of the ongoing need to audit the outcomes of the use of LCIG in a cohort of patients either unsuitable or not sufficiently helped by apomorphine and/or DBS and thus justify both the invasive nature and expense, we have examined the outcome of LCIG use in our own service, using the PDQ39 measure of quality of life and its subscores, as well as diary data regarding periods spent “On,” “Off,” “Asleep,” and “On with LID.”

## 2. Methods

All patients included in this report met the Queen Square Brain Bank for Neurological Disorders criteria for the diagnosis of PD [12] and were in the advanced stages of the disease with severely disabling motor fluctuations and dyskinesias despite optimal oral PD treatment. All patients had been treated with subcutaneous apomorphine but nevertheless had persistent disabling motor fluctuations and dyskinesias and had been considered for DBS surgery by an expert multidisciplinary DBS team. Two individuals had already previously undergone DBS surgery, while 3 were considered too old, 4 had cognitive or psychiatric contraindications, and 3 preferred to avoid surgery. Baseline severity of PD was assessed using an L-dopa challenge and scores recorded using the Unified Parkinson's disease rating scale. All patients completed diaries to document the clinical state of their PD throughout the waking day (6 am until midnight) and completed the PDQ39 questionnaire.

LCIG was started on an inpatient basis. Each patient had a nasojejunal (NJ) tube inserted under endoscopic guidance to allow a trial period to assess both the tolerability and clinical improvement that could be achieved with LCIG delivered via a CADD pump. All oral PD medications were stopped during this period except for the use of oral L-dopa that could be taken at bedtime when the pump was discontinued. The dose of LCIG required to optimise PD control was titrated incrementally over the first 3 days. The NJ tube was then converted to a 15-F-diameter percutaneous endoscopic gastrostomy with 8F jejunal extension (PEG-J) tube in patients showing clear evidence of benefit. NJ and PEG-J insertion was performed by a single endoscopist (GJW), and PEG-J insertion was performed under endoscopic visualisation and X-ray imaging.

Following discharge from hospital, patients were seen in the outpatient clinic at 1 month, 3 months, 6 months, and 12 months. They were given PD motor diaries and the PDQ39 to complete at each visit. All data were checked for distribution normality using the Shapiro-Wilk test and then paired *t*-tests used to compare baseline scores with scores at latest followup on LCIG.

## 3. Results

A description of the 12 patients started on LCIG is presented in Table 1. 

In eleven patients it was decided to proceed in replacing the NJ tube by a PEG-J tube given the evidence of improvement in PD control. In patient 4, the NJ assessment period was prolonged to 2 weeks to properly establish whether LCIG could lead to an improvement in cognitive impairment via prolonged withdrawal of dopamine agonist medication. In this individual there was clear evidence of persisting behavioural problems associated with lack of insight (presumably reflecting widespread cortical Lewy body pathology) despite LCIG, and it was decided on clinical grounds not to proceed with LCIG therapy. 

**Figure 1:**
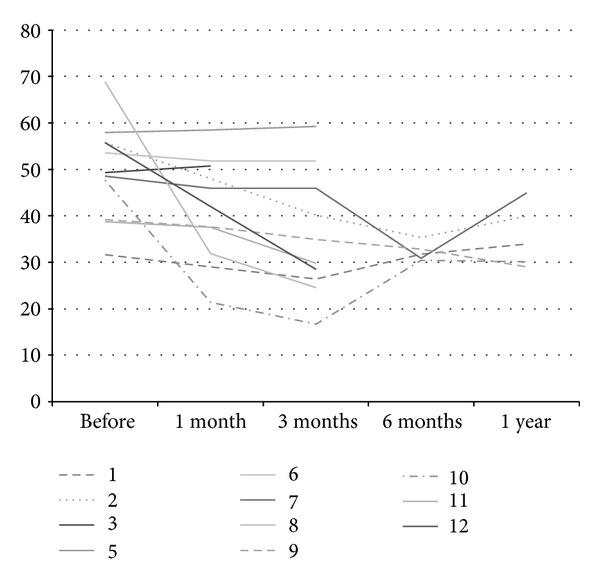
PDQ39 summary index in 11 patients exposed to Duodopa with longitudinal followup.

**Figure 2:**
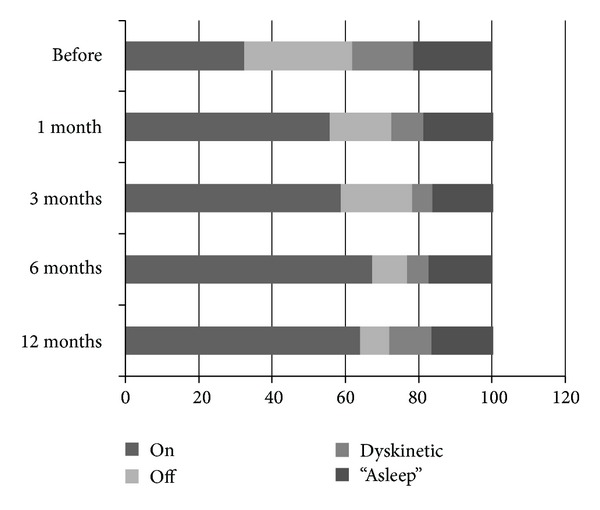
Summary data from diaries of patients persisting with LCIG treatment.

The PDQ39 scores are presented in Table 2 and the PDQ39 summary index in Figure 1. In the comparison between baseline and last followup, while still using Duodopa, there was a mean significant improvement seen in the PDQ39 summary index as well as the subscores of “Mobility,” “Sense of Stigma,” and “Cognition.” Inspection of individual data (Figure 1) shows evidence of improvement in PDQ39 summary index in 6 individuals while in 5 individuals, quality of life was subjectively unchanged. The diary data are presented in Figure 2 showing an approximate doubling of the time spent on without dyskinesia and reduction in Off time, dyskinetic time, and time asleep during the daytime diary period. Among the 5 individuals without improvement in quality of life, 3 were nevertheless substantially better based on diary evaluations and therefore may have had unrealistic expectations of improvement in nonmotor symptoms. Two individuals had no improvement on diary evaluation or PDQ39, of these 1 had an LRRK2 mutation and previously had responded poorly to DBS, and the other had the lowest severity of disease of the group and stopped LCIG in order to seek out “experimental disease-modifying therapies.” Mean comparisons of baseline and latest follow-up scores for the diary data are presented in Table 3.

### 3.1. Adverse Events

Of the 11 patients who had PEG-J tubes sited, no procedure-related or early major complications were seen (e.g., bleeding, perforation, and systemic sepsis). However, 3 did not continue LCIG for greater than 3 months (2 stopped because of difficulties with the PEG-J tube, and 1 person died unexpectedly 3 months after introduction of LCIG therapy. Although no postmortem occurred, the death was not considered related to LCIG treatment. Of the remaining 8 patients, 4 have had recurrent minor problems with PEG-J tube (resiting jejunal extension x9 occasions, replacement of PEG tube x3 occasions, blocked jejunal tube x3 occasions, broken PEG flange x1 occasion, cracked PEG bung x1 occasion). A further patient died from Ca colon but continued to have effective treatment with LCIG up until his death. Increased impulsivity was observed in 2 patients, including reckless mobilisation despite postural instability in 1 individual and impulsive spending in the other. One individual experienced hypergranulation around the PEG-J site requiring topical steroid application. No patient reported symptoms of neuropathy.

## 4. Discussion

Very few data have been published regarding the outcome of Duodopa among patients in the UK [13, 14] possibly because of the widespread use of subcutaneous Apomorphine and Deep Brain Stimulation. These LCIG data are broadly consistent with those previously reported from other series in which prior apomorphine use had not been so frequent [3, 4], and in the largest series that used retrospectively collected data [15]. Our prospectively collected diary data and PDQ39 scores suggest that there is a significant mean improvement in the amount of good quality ON time in well-selected patients treated with LCIG, even after apomorphine/DBS failure or nonsuitability. 

Some of the beneficial effects of LCIG relate to the ability to provide continuous plasma levels of Levodopa, which lead to a more consistent delivery of dopa to the brain. Other beneficial effects, for example, PDQ39-cognition, may well also relate to withdrawal of dopamine agonist use. We did not find any evidence for improvement in some of the PDQ39 subindices, for example, “Bodily discomfort,” suggesting that not all aspects of this subindex relate to dopa responsive symptoms and also not surprisingly in the subindex—“Social Support.” We have not collected data to establish any positive economic impact of LCIG use, although this has been attempted previously using published efficacy data in comparison to the known costs of LCIG [16]. Some of the costs of LCIG can be offset against dopamine agonist discontinuation (apomorphine, ropinirole, pramipexole, or rotigotine).

Despite the improvements seen, adverse events are not uncommon, and patients require regular and ongoing support from our PD specialist nurses, the gastroenterology team, as well as support provided directly from the Abbott commercial team. In addition to the clinical assessments routinely performed to judge the merits of the use of this treatment, there appears to be a degree of self-selection, in that patients that do not receive a substantial benefit from the use of this therapy seem not to wish to persist with it in the long term, largely due to the not infrequent complications associated with the PEG-J tube. Maximising the benefits that can be received by the “good responding” patients requires a close and effective partnership between the PD and gastroenterology services.

The optimal selection of patients for Duodopa treatment requires personal experience of its potential benefits as well as experience of the use of the alternative treatments for the motor complications of advanced PD, namely, apomorphine and DBS. All 3 approaches can be associated with adverse events and an open, balanced discussion of these without engendering fear is essential in guiding a patient towards the correct treatment choice. In the UK LCIG is only available for patients who have had been previously considered for, or failed, treatment with apomorphine, and patient preference remains a major factor to be considered in subsequent treatment decisions. Our own experience suggests that options such as LCIG may be helpful in certain individuals even after DBS.

 Prescription of LCIG therapy in the UK is considered following receipt of an “Individual funding request” to a patient's respective Primary Care Trust under the “exceptional circumstances” policy. The supporting evidence regarding the beneficial effects of LCIG and an individual's circumstances is considered and inevitably weighed up against the high cost of the drug. Each patients' LCIG treatment costs must be added to the costs of the PEG-J procedure(s) and any necessary follow-up gastroenterology clinic visits. Given this expense, and the arbitrary nature by which patients are judged to be exceptional, the decision to approve or reject funding applications varies greatly. 

In these circumstances, advice can be usefully provided by consensus decisions reached by expert committees (e.g., via the National Institute for Health and Clinical Excellence (NICE)); however given the timing of the most recent review of treatment guidelines for advanced PD, LCIG is yet to be appraised by NICE (although it is hoped that this will happen in the near future). 

From the perspective of the healthcare provider, we believe that the optimal delivery of appropriately tailored treatment to an individual requires input from regional neurological centres with experience of the use of all 3 treatments. Services need not be delivered solely in these “hub” centres; however each patient judged to be a good candidate ought to have had adequate information regarding the use of all 3 approaches. Rather than the current “postcode lottery,” a ceiling on the number of LCIG treatments that can be initiated by each “Specialist centre” each year would seem to us a more equitable and practical way of delivering the best available treatment in advanced PD while ensuring that financial constraints in public health service budgets are not overwhelmed.

## Figures and Tables

**Table 1:** Description of patient initiated on LCIG therapy at our centre.

Patient number	Age at onset of PD (yrs)	Genetic cause	UPDRS3 Off/On	Age started LCIG (yrs)	Length of LCIG treatment to date (months)
1	28		88/30	59	47
2	30		74/37	59	46
3	28	Parkin	62/33	63	3—deceased
4	38		67/43	66	Judged unsuitable during NJ phase
5	48		54/20	67	3—stopped
6	49	LRRK2	78/33	68	24—deceased (Ca colon)
7	48		66/50	72	26
8	45		44/5	63	23
9	44		57/22	66	21
10	58		62/	72	21
11	55		50/30	72	3—stopped
12	43	Parkin	51/31	65	5

**Table 2:** Mean (SD) and *P* values of paired *t*-tests of scores at baseline and at latest followup.

	Before Duodopa (*n* = 11) Mean (SD)	1 month (*n* = 11) Mean (SD)	3 months (*n* = 10) Mean (SD)	Latest (*n* = 11) Mean (SD)	Before versus latest *P* value
Mobility	76.4 (19.9)	62.8 (24.0)	56.8 (29.5)	56.3 (13.0)	0.04
ADL	62.1 (17.1)	55.2 (24.2)	50.0 (27.8)	52.6 (24.9)	0.23
Emotional well-being	45.8 (13.1)	40.7 (7.8)	38.3 (9.4)	38.6 (9.1)	0.13
Sense of stigma	33.5 (16.8)	24.9 (15.8)	16.3 (15.4)	19.3 (17.7)	0.03
Social support	22.0 (20.5)	14.3 (13.5)	17.5 (9.2)	18.8 (14.3)	0.32
Cognition	52.8 (20.6)	37.1 (17.8)	32.5 (12.7)	32.4 (10.4)	0.02
Communication	53.0 (17.6)	40.6 (22.9)	35 (24.2)	40.9 (25.1)	0.19
Bodily discomfort	52.3 (23.6)	53.8 (22.8)	40 (25.4)	49.2 (28.0)	0.59
Summary index	49.7 (10.4)	41.3 (11.1)	35.8 (13.3)	38.7 (11.2)	0.02

**Table 3:** Mean percentage time spent “Off”, “On,” “Dyskinetic,” or “Asleep.”

	Baseline Mean (SD)	Latest follow-up Mean (SD)	*P* value
Diary On time	32.4 (15.5)	56.2 (23.5)	0.01
Diary Off time	29.4 (13.2)	16.7 (22.2)	0.06
Diary Dyskinetic time	16.6 (18.6)	8.2 (10.3)	0.22
Asleep	21.7 (9.3)	18.5 (7.2)	0.26
